# Transplantation of vascular cells derived from human embryonic stem cells contributes to vascular regeneration after stroke in mice

**DOI:** 10.1186/1479-5876-6-54

**Published:** 2008-09-30

**Authors:** Naofumi Oyamada, Hiroshi Itoh, Masakatsu Sone, Kenichi Yamahara, Kazutoshi Miyashita, Kwijun Park, Daisuke Taura, Megumi Inuzuka, Takuhiro Sonoyama, Hirokazu Tsujimoto, Yasutomo Fukunaga, Naohisa Tamura, Kazuwa Nakao

**Affiliations:** 1 Department of Medicine and Clinical Science, Kyoto University Graduate School of Medicine, Japan Department of Medicine and Clinical Science, Kyoto University Graduate School of Medicine, 54 Shogoin Kawahara-cho, Sakyo-ku, Kyoto, 606-8507, Japan; 2 Department of Internal Medicine, Keio University School of Medicine 35 Shinanomachi, Shinjuku-ku Tokyo 160-8582, Japan

## Abstract

**Background:**

We previously demonstrated that vascular endothelial growth factor receptor type 2 (VEGF-R2)-positive cells induced from mouse embryonic stem (ES) cells can differentiate into both endothelial cells (ECs) and mural cells (MCs) and these vascular cells construct blood vessel structures in vitro. Recently, we have also established a method for the large-scale expansion of ECs and MCs derived from human ES cells. We examined the potential of vascular cells derived from human ES cells to contribute to vascular regeneration and to provide therapeutic benefit for the ischemic brain.

**Methods:**

Phosphate buffered saline, human peripheral blood mononuclear cells (hMNCs), ECs-, MCs-, or the mixture of ECs and MCs derived from human ES cells were intra-arterially transplanted into mice after transient middle cerebral artery occlusion (MCAo).

**Results:**

Transplanted ECs were successfully incorporated into host capillaries and MCs were distributed in the areas surrounding endothelial tubes. The cerebral blood flow and the vascular density in the ischemic striatum on day 28 after MCAo had significantly improved in ECs-, MCs- and ECs+MCs-transplanted mice compared to that of mice injected with saline or transplanted with hMNCs. Moreover, compared to saline-injected or hMNC-transplanted mice, significant reduction of the infarct volume and of apoptosis as well as acceleration of neurological recovery were observed on day 28 after MCAo in the cell mixture-transplanted mice.

**Conclusion:**

Transplantation of ECs and MCs derived from undifferentiated human ES cells have a potential to contribute to therapeutic vascular regeneration and consequently reduction of infarct area after stroke.

## Background

Stroke, for which hypertension is the most important risk factor, is one of the common causes of death and disability in humans. It is widely considered that stroke patients with a higher cerebral blood vessel density show better progress and survive longer than patients with a lower vascular density. Angiogenesis, which has been considered to the growth of new capillaries by sprouting of preexisting vessels through proliferation and migration of mature endothelial cells (ECs), plays a key role in neovascularization. Various methods for therapeutic angiogenesis, including delivery of angiogenic factor [1,2] or cell transplantation [3-5], have been used to induce collateral blood vessel development in several animal models of cerebral ischemia. More recently, an alternative paradigm, known as postnatal vasculogenesis, has been shown to contribute to some forms of neovascularization. In vasculogenesis, endothelial progenitor cells (EPCs), which have been recognized as cellular components of the new vessel structure and reserved in the bone marrow, can take an important part in tissue neovascularization after ischemia [6]. Previous reports demonstrated that transplantation of mouse bone marrow cells after cerebral ischemia increased the cerebral blood flow partially via the incorporation of EPCs into host vascular structure as vasculogenesis [4]. However, because the population of EPCs in the bone marrow and in the peripheral blood has been revealed to be very small [7], it is now recognized to be difficult to prepare enough EPCs for the promotion of therapeutic vaculogenesis after ischemia.

We previously demonstrated that VEGF-R2-positive cells induced from undifferentiated mouse embryonic stem (ES) cells can differentiate into both VE-cadherin-positive endothelial cells (ECs) and αSMA-positive mural cells (MCs), and these vascular cells construct blood vessel structures [8]. We have also succeeded that after the induction of differentiation on OP9 feeder layer, VEGFR-2-positive cells derived from not only monkey ES cells [9] but human ES cells [10], effectively differentiated into both ECs and MCs. Next, we demonstrated that VE-cadherin^+^VEGF-R2^+^TRA-1^-^cells differentiated from human ES cells on day 10 of differentiation, which can be considered as ECs in the early differentiation stage, could be expanded on a large scale to produce enough number of ECs for transplantation [10]. Moreover, we also succeeded in expanding not only ECs but also MCs derived from these ECs in the early differentiation stage in vitro.

In the present study, we examined whether ECs and MCs derived from human ES cells could serve as a source for vasculogenesis in order to contribute to therapeutic neovascularization and to neuroprotection in the ischemic brain.

## Methods

### Preparation of human ECs and/or MCs derived from human ES cells

**Figure 1:**
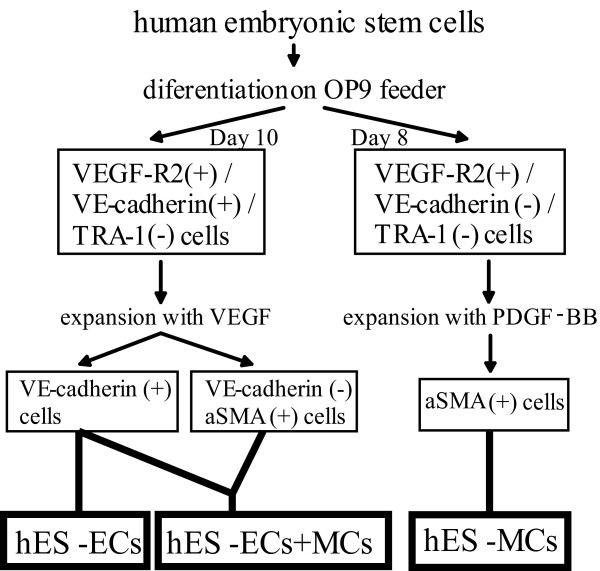
Schematic representation of preparation of the transplanted vascular cells differentiated from human ES cells.

Maintenance of human ES cell line (HES-3) was described previously [10]. We plated small human ES colonies on OP9 feeder layer to induce differentiation into ECs and MCs [10]. On day 10 of differentiation, VE-cadherin^+^VEGF-R2^+^TRA-1^- ^cells were sorted with a fluorescence activator cell sorter (FACSaria; Becton Dickinson). Monoclonal antibody for VEGF-R2 was labeled with Alexa-647 (Molecular Probes). Monoclonal antibody for TRA1-60 (Chemicon) was labeled with Alexa-488 (Molecular Probes) and anti VE-cadherin (BD Biosciecnces) antibody was labeled with Alexa 546 (Molecular Probes). After sorting the VE-cadherin^+^VEGFR-2^+^TRA-1^- ^cells on day 10 of differentiation, we cultured them on type IV collagen-coated dishes (Becton Dickinson) with MEM in the presence of 10% fetal calf serum (FCS) and 50 ng/ml human VEGF165 (Peprotech) and expanded these cells. After five passages in culture (= approximately 30 days after the sorting), we obtained the expanded cells as a mixture of ECs and MCs derived from human ES cells (hES-ECs+MCs). The cell mixture was composed of almost the same number of ECs and MCs. We resorted the VE-cadherin^+ ^cells from these expanded cells to obtain ECs for transplantation (Figure 1). The ECs derived from human ES cells (hES-ECs) were labeled with CM-Dil (Molecular Probes) before the transplantation.

After sorting VE-cadherin^-^VEGFR-2^+^TRA-1^- ^cells on day 8 of differentiation, we cultured these cells on type IV collagen-coated dishes by five passages (= approximately 40 days after the sorting) in the presence of 1% FCS and PDGF-BB (10 ng/ml) (PeproTech) to obtain only MCs derived from human ES cells (hES-MCs) for the transplantation (Figure 1). On the day of transplantation, these cells were washed with PBS twice and harvested with 0.05% trypsin and 0.53 mmol/L EDTA (GIBCO) for 5 minutes. Each cells used for the transplantation was suspended in 50 ul PBS.

### Preparation of human mononuclear cells

We performed the transplantation of human mononuclear cells (hMNCs), which contain a very small population of EPCs (≦ 0.02%) [7], to examine the non-specific influences due to the cell transplantation itself. The hMNCs were prepared from 10 ml samples of peripheral blood of healthy volunteers. Each sample was diluted twice with PBS and layered over 8 ml of Ficoll (Biosciences). After centrifugation at 2500 g for 30 minutes, the mononuclear cell layer was harvested in the interface and resuspended in PBS (3 × 10^6 ^cells/50 ul) for the transplantation.

### Immunohistochemical examination of cultured cells

Staining of cultured cells on dishes at 5^th ^passage was performed as described elsewhere [8,10]. Monoclonal antibodies for alpha smooth muscle actin (αSMA) (Sigma), human CD 31 (BD Biosciecnces) and calponin (Dako Cytomation) were used.

### Middle cerebral artery occlusion (MCAo) model and cell transplantation

We used adult male C57 BL6/J mice weighing 20–25 g for all our experiments, and all of them were anesthetized with 5% halothane and maintained 1% during the experiments. We induced transient left middle cerebral artery occlusion (MCAo) for 20 min as previously described [11]. Briefly, a 8-0 nylon monofilament coated with silicone was inserted from the left common carotid artery (CCA) via the internal carotid artery to the base of the left MCA. After the occlusion for 20 minutes, the filament was withdrawn and intra-arterial injection of hES-derived vascular cells was performed through the left CCA. We prepared four groups of the transplanted cells; Group1: PBS (50 ul), Group 2: hMNCs (3 × 10^6 ^cells), Group 3: hES-ECs (1.5 × 10^6 ^cells), Group 4: hES-MCs (1.5 × 10^6 ^cells), Group 5: hES-ECs+MCs (3 × 10^6 ^cells). After transplantation, the distal portion of CCA was ligated. All animals were immunosuppressed with cyclosporin A (4 mg/kg, ip) on day 1 before the transplantation, postoperative day 1–7, 10, 14, and 21. Experimental procedures were performed in accordance with Kyoto University guidelines for animal experiments.

### Assessment for cerebral blood flow after the transplantation

We measured the cerebral blood flow (CBF) just before the experiments (= day 0) and on day 4 and 28 after MCAo by mean of a Laser-Doppler perfusion imager (LDPI, Moor Instruments Ltd.). During the measurement, each mouse was anesthetized with halothane and the room temperature was kept at 25–27°C. The ratio of blood flow of the area under MCA in the ipsilateral side to the contralateral side was calculated as previously described [11].

### Immunohistochemical examination of the ischemic striatum

The harvested brains were subjected to immunohistochemical examination using a standard procedure as previously described [12]. In all of our examination, free-floating 30-μm coronal sections at the level of the anterior commisure (= the bregma) were stained and examined with a confocal microscope (LSM5 PASCAL, Carl Zeiss). Sections were subjected to immunohistochemical analysis with the antibodies for human PECAM-1 (BD Biosciecnces, 1:100), mouse PECAM-1 (BD Bioscience, 1:100), human HLA-A, B, C (BD Biosciecnces, 1:100), αSMA (BD Biosciecnces, 1:100), Neu-N (Chemicon, 1:200), and single stranded DNA (Dako Cytomation, 1:100).

In our model of MCAo, the infarct area was confined to the striatum. The ischemic striatum at the level of the anterior commisure from each mouse was photographed on day 28 after MCAo. The procedure of the quantification of vascular density was carried out as described in Yunjuan Sun et al. [13] with slight modification. Vascular density in the ischemic striatum was examined at ×20 magnification, by quantifying the ratio of the pixels of human and/or mouse PECAM-1-positive cells to 512 × 512 pixels in that field: the ratio was expressed as %area. The number of transplanted MCs detected in the ischemic core at ×20 magnification was calculated. To identify localization of transplanted ECs or MCs, the fields in the ischemic striatum were photographed at ×63 magnification. The infarct area (mm^2^/field/mouse) at the level of the bregma was defined and quantified as the lesion where Neu-N immunoreactivity disappeared in the striatum at ×5 magnification as previously described [11,14]. The measurement of infarct volumes was carried out as described in Sakai T. et al. [14] with slight modification. Another saline- and EC+MC-injected groups were sacrificed on day 28 after MCAo. For the measurements of the infarct volume, 5 coronal sections (approximately -1 mm, -0.5 mm, ± 0 mm, +0.5 mm and +1 mm from the bregma) were prepared from each mouse and each infarct area (mm^2^) was measured. And then, the infarct area was summed among slices and multiplied by slice thickness to provide infract volume (mm^3^). To calculate apoptotic cells, the number (cells/mm^2^/mouse) of single stranded DNA (ss-DNA)^+ ^cells in one field in the ischemic core from each mouse in the saline- or hES-ECs+MCs-injected group was quantified at ×20 magnification on day 14 after MCAo.

### Neurological Functional test

We used the rota-rod exercise machine for the assessment of the recovery of impaired motor function after MCAo. This accelerating rota-rod test was carried out as described in A.J. Hunter et al. [15] with slight modification. Each mouse was trained up to be able to keep running on the rotating rod over 60 seconds at 9 round per minutes (rpm) (2^th ^speed). After the training was completed, we placed each mouse on the rod and changed the speed of rotation every 10 seconds from 6 rpm (1^st ^speed) to 30 rpm (5^th ^speed) over the course of 50 seconds and checked the time until the mouse fell off. The exercise time (seconds) on the rota-rod for each mouse was recorded just before the experiments (= day 0) and on day 7 and 28 after MCAo.

### Analysis of mRNA expression of angiogenic factors

Cultured human aortic smooth muscle cells (hAoSMC) (Cambrex, East Rutherford, NJ) were used for control. Total cellular RNA was isolated from hES-MCs and human aortic smooth muscle cells (hAoSMC) (Cambrex, East Rutherford, NJ) with RNAeasy Mini Kit (QIAGEN K.K., Tokyo, Japan). The mRNA expression was analyzed with One Step RNA PCR Kit (Takara, Out, Japan). The primers used were as follows: human vascular endothelial growth factor (VEGF, Genbank accession No.X62568), 5'-AGGGCAGAATCATCACGAAG-3' (forward) and 5'-CGCTCCGTCGAACTCAATTT-3' (reverse); human basic fibroblast growth factor (bFGF, Genbank accession No.M27968), AGAGCGACCCTCACATCAAG (forward) and TCGTTTCAGTGCCACATACC (reverse); human hepatic growth factor (HGF, Genbank accession No.X16323), 5'-AGTCTGTGACATTCCTCAGTG-3' (forward) and 5'-TGAGAATCCCAACGCTGACA-3' (reverse); human platelet-derived growth factor (PDGF-B, Genbank accession No.X02811), 5'-GCACACGCATGACAAGACGGC-3' (forward) and 5'-AGGCAGGCTATGCTGAGAGGTCC-3' (reverse); and GAPDH (Genbank accession No.M33197), 5'-TGCACCACCAACTGCTTAGC-3' (forward) and 5'-GGCATGGACTGTGGTCATGA-3' (reverse). Polymerase chain reactions (PCR) were performed as described in the manufacturer's protocols.

### Measurement of angiogenic factors in hES-MCs-conditioned media

After 1 × 10^6 ^cells of hES-MC or hAoSMC were plated on 10 cm type IV collagen-coated dishes and incubated with 5 ml media (αMEM with 0.5% bovine serum) for 72 hours, the concentration of human VEGF, bFGF and HGF were measured by SRL, Inc. (Tokyo, Japan).

### Statistical analysis

All data were expressed as mean ± standard error (S.E.). Comparison of means between two groups was performed with Student's t test. When more than two groups were compared, ANOVA was used to evaluate significant differences among groups, and if there were confirmed, they were further examined by means of multiple comparisons. Probability was considered to be statistically significant at P < 0.05.

## Results

### Preparation and characterization of transplanted cells derived from human ES cells

**Figure 2:**
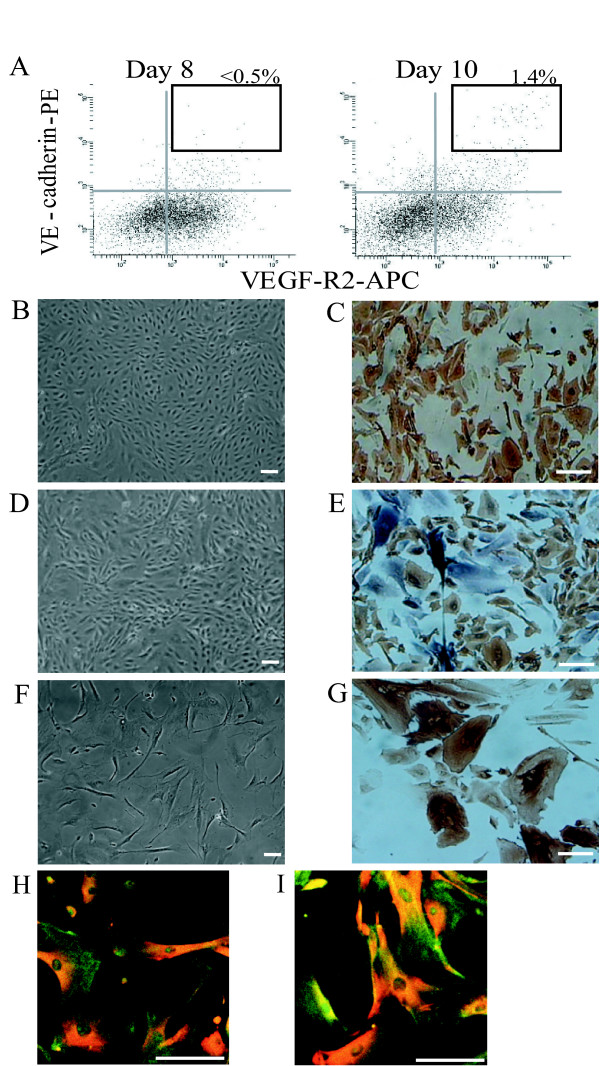
**Characterization of the transplanted vascular cells derived from human ES cells (HES-3)**. A, Flow cytometric analysis of VE-cadherin and VEGF-R2 expression on human ES cells during differentiation on an OP9 feeder layer. VE-cadherin^+^VEGF-R2^+^TRA-1^- ^cells are indicated by the boxed areas. B, Morphology of the VE-cadherin^+ ^cells (= hES-ECs) resorted from expanded VE-cadherin^+^VEGF-R2^+^TRA-1^- ^cells at 5^th ^passage. C, Immunostaining for human PECAM-1 (brown) of hES-ECs. D, Morphology of the expanded VE-cadherin^+^VEGF-R2^+^TRA-1^- ^cells at 5^th ^passage (= hES-ECs+MCs). E, Double immunostaining for human PECAM-1 (brown) and αSMA (purple) on hES-ECs+MCs. F, Morphology of the cells (= hES-MCs) expanded from VE-cadherin^-^VEGF-R2^+^TRA-1^- ^cells on day 10 of differentiation with PDGF-BB and 1% FCS up to 5^th ^passage. G, Immunostaining for αSMA (brown) of hES-MCs. H-I, Immunostaining for αSMA (green) and calponin (red) of hAoSMCs (H) and hES-MCs (I). Scale bar: 50 μm.

We induced differentiation of human ES cells in an in vitro two-dimensional culture on OP9 stromal cell line and examined the expression of VEGF-R2, VE-cadherin and TRA-1 during the differentiation. While the population of VE-cadherin^+^VEGF-R2^+^TRA-1^- ^cells was not detected (< 0.5%) before day 8 of differentiation, it emerged and accounted for about 1–2% on day10 of differentiation (Figure 2A). As we previously reported, these VE-cadherin^+^VEGF-R2^+^TRA-1^- ^cells on day 10 of differentiation were also positive for CD34, CD31 and eNOS [10]. Therefore, we used the term 'eEC' for these ECs in the early differentiation stage. We sorted and expanded these eECs in vitro. These eECs were cultured in the presence of VEGF and 10% FCS and expanded by about 85-fold after 5 passages. The expanded cells at 5^th ^passage were constituted with two cell fractions. One of these cells was VE-cadherin^+ ^cells (35–50%), which were positive for other endothelial markers, including, CD31 (Figure 2B–E) and CD34 [10], indicating that cell differentiation stage had been retained. The other was VE-cadherin^- ^cells (50–65%), which were positive for αSMA and considered to differentiate into MCs (Figure 2D–E). We sorted the fraction of VE-cadherin^-^VEGF-R2^+^TRA-1^- ^cells, which appeared on day 8 of differentiation and were positive for platelet derived growth factor receptor type β (PDGFR-β) [10], and expanded these cells for induction to MC in the presence of PDGF-BB and 1% FCS. At passage 5, all of the expanded cells effectively differentiated into αSMA-positive MCs (Figure 2F–G).

### Assessment of cerebral blood flow recovery in the infarct area after the transplantation

**Figure 3:**
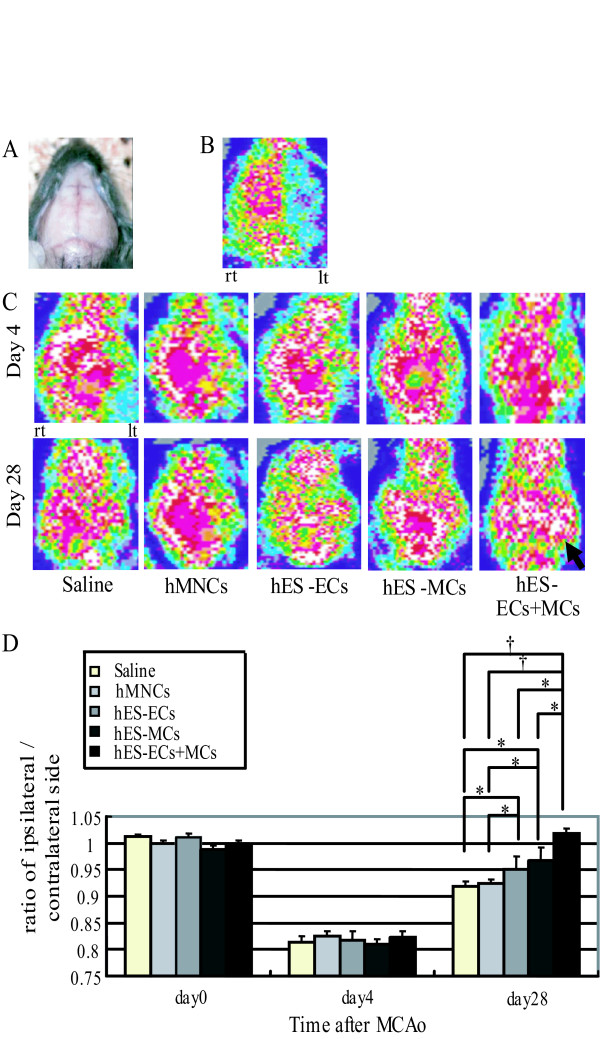
**Effects of the transplanted vascular cells on the CBF in the ipsilateral side**. A-C: LDPI analysis of the CBF by LDPI evaluated in mice with the scalp removed (A). Flowmetric analysis of the CBF in the ipsilateral side (= left side: lt) during MCA-occlusion (B). The CBF in the ipsilateral and contralateral side in the five groups on day 4 and 28 after MCAo (C). An arrow indicates the lesion in the hES-EC+MC-injected group where the CBF clearly increased up to or rather than the corresponding area in the contralateral side. Red or white indicates higher flow than blue or green. D, Quantitative analysis of the CBF ratio of the ipsilateral/contralateral side just before the experiments (= day 0) and on day 4 and 28 after MCAo. * *P *< 0.05, † *P *< 0.01.

As shown in Figure 3B, the cerebral blood flow in the ipsilateral side decreased by approximately 80% compared to that in the contralateral side during MCAo and the area with the suppressed blood flow was corresponded to the area under MCA. In the 5 groups, the CBF ratio on day 4 decreased by about 20% compared to that of the contralateral side due to ligation of the left CCA after the transplantation. Then, we assessed the recovery of the CBF in the ipsilateral side from this time point. Apparent difference in the CBF in the ipsilateral side was not observed among the 5 groups on day 4 after MCAo. However, the blood flow of the ipsilateral side in the hES-EC+MC-injected group, especially pointed out by the arrow, clearly increased up to or rather than the corresponding area in the contralateral side on day 28 after MCAo, compared to other 4 groups (Figure 3C). On day 28, the CBF ratio of the saline- and hMNC-injected group were similar (Figure 3D), while that of hES-EC-injected group increased significantly compared to that of these two groups (saline: 0.919 ± 0.010, n = 12. hMNCs: 0.925 ± 0.008, n = 15. hES-ECs: 0.952 ± 0.025, n = 7. *P *< 0.05). The CBF ratio of the hES-MC-injected group (0.968 ± 0.023, n = 7. *P *< 0.05) increased significantly compared to that of the saline- or hMNCs-injected groups on day 28, while that of the hES-EC+MC-injected group (1.018 ± 0.009: n = 13) increased significantly compared to not only that of the saline- or hMNCs-injected groups (*P *< 0.001), but also that of the hES-EC- or hES-MC-injected group (*P *< 0.01).

### Localization of transplanted vascular cells derived from human ES cells and the vascular density in the infarct area after the transplantation

**Figure 4:**
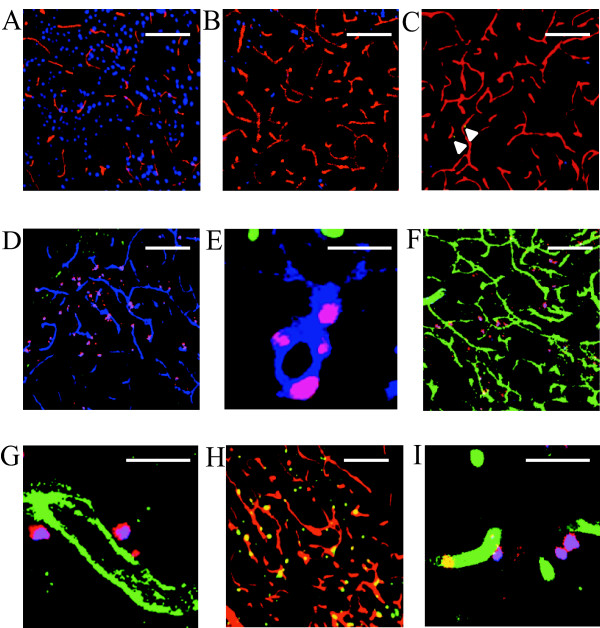
**Histological examination of the vasculature in the non-ischemic and ischemic striatum on day 28 after MCAo**. A-C: Immunostaining of mouse PECAM-1 (red)/Neu-N (blue) in the non-ischemic striatum (A), and the ischemic striatum in saline (B)-and hMNC (C)-injected mice. Arrows show human PECAM-1^+ ^(green) cells in the ischemic striatum in the hMNC-injected group. D-E: Representative fluorescent photographs of the ischemic striatum stained for mouse PECAM-1 (blue), Neu-N (green) and CM-DiI (red) in hES-EC-injected mice. F-G: Immunostaining of αSMA (blue)/mouse PECAM-1 (green)/human HLA-A,B,C (red) in the ischemic striatum in the hES-MC-injected mice. Human HLA positive and αSMA positive hES-MCs were shown as purple (red+blue) cells. H, Immunostaining of mouse PECAM-1 (red)/Neu-N (blue)/human Pecam-1 (green) in the ischemic striatum in the hES-EC+MC-injected groups. I, Localization of transplanted hES-ECs+MCs in the ischemic striatum stained for αSMA (blue)/mouse PECAM-1 (green)/human HLA-A,B,C (red). A-D/F/H, scale bar: 100 μm, ×20 magnification. E/G/I, scale bar: 20 μm, ×63 magnification.

In the saline- and hMNCs-injected groups, the vascular density of host capillary quantified by mouse PECAM-1 immunoreactivity in the ischemic striatum (Figure 4B, C) was higher than that in the non-ischemic striatum (Figure 4A). In hMNCs-injected group, few human PECAM-1 positive cells were observed in the ischemic striatum (Figure 4C) and these cells were not found in the non-ischemic striatum. In the hES-EC-injected group, many DiI positive hES-ECs were observed in the infarct area (Figure 4D) and incorporated into the host capillaries (Figure 4E). In the hES-MC-injected group, both αSMA and human HLA positive cells (23.1 ± 2.0 counts/field: n = 7) were detected in the infarct area (Figure 4F) and localized in the conjunction with mouse endothelial tubes (Figure 4G). Compatible with these results, in the hES-EC+MC-injected group, many human PECAM-1 positive cells were detected in the host capillaries (Figure 4H) while transplanted MCs (21.7 ± 1.8 counts/field: n = 6) surrounded the capillaries in the infarct area, similarly to those in the hES-MCs-injected group (Figure 4I).

**Figure 5:**
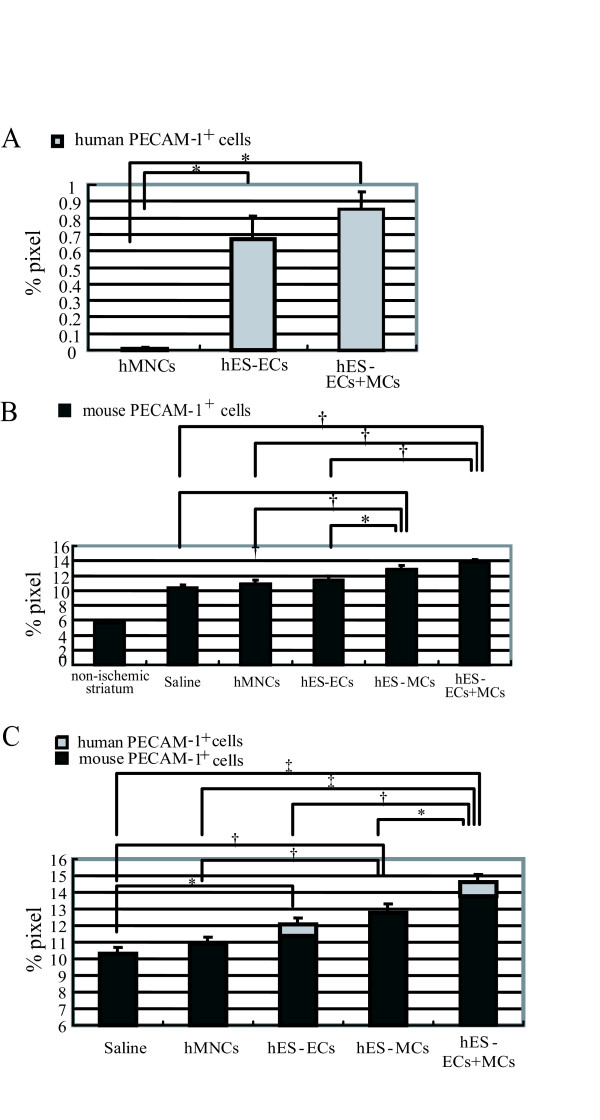
**Evaluation of vascular regeneration in the striatum on day 28 after stroke in the five groups**. A, Quantification of the density of human PECAM-1^+ ^cells (%area) in the ischemic striatum in hMNC-, hES-EC- and hES-EC+MC-injected groups. * *P *< 0.0001. B, Quantitative analysis of the density of mouse PECAM-1^+ ^cells (%area) in the non-ischemic striatum and in the ischemic striatum in five groups. * *P *< 0.05, † *P *< 0.01. C, Quantification of the total density of human and mouse PECAM-1^+ ^cells (%area) in the ischemic striatum in five groups. * *P *< 0.05, †*P *< 0.01, ‡ *P *< 0.001.

In the ischemic striatum, the density (%area) of human PECAM-1 positive cells was 0.05 ± 0.01% in the hMNC-injected group (n = 11), 0.66 ± 0.11% in the hES-EC-injected group (n = 7, *P *< 0.0001 vs hMNCs) and 0.85 ± 0.12% in the hES-EC+MC-injected group (n = 11, *P *< 0.0001 vs hMNCs) (Figure 5A). As shown in Figure 5B, there was no significant difference in the densities of mouse PECAM-1 positive cells among the saline- (10.3 ± 0.4%: n = 11), hMNC- (10.9 ± 0.3%: n = 11) and hES-EC- (11.4 ± 0.4%: n = 7) injected groups, although the densities were significantly higher than that in the non-ischemic striatum (5.6 ± 0.2%: n = 5). In hES-MC- (13.2 ± 0.5%: n = 7, *P *< 0.01 vs control, *P *< 0.05 vs hES-ECs) or hES-EC+MC- (13.8 ± 0.4%: n = 11, *P *< 0.01 vs control and hES-ECs) injected group, a significant increase in the density of mouse PECAM-1 positive cells was observed. The total vascular density estimated by summing up human and mouse PECAM-1 positive area (12.2 ± 0.6%, *P *< 0.05) in the hES-EC-injected group was significantly higher compared to that in the saline-injected group. Moreover, the total vascular density in the hES-EC+MC-injected group (14.7 ± 0.6%) was markedly higher compared to those in the other four groups (*P *< 0.001 vs control, *P *< 0.01 vs hES-ECs, *P *< 0.05 vs hES-MCs) (Figure 5C).

### Analysis of the infarct size and apoptosis in the ipsilateral side after the transplantation

**Figure 6:**
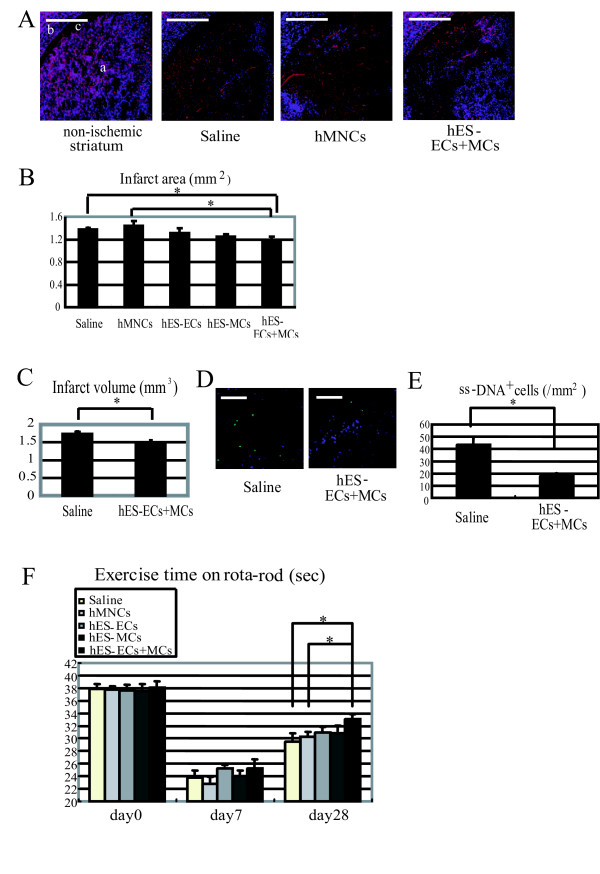
**Effects of the transplanted cells on neuroprotection and recovery of impaired motor function after MCAo**. A-B, Representative fluorescent photograph in non-ischemic and ischemic striatum. a, striatum; b, cortex; c, external capsule. The area where Neu-N expression was lost in the striatum in the saline-, hMNC- and hES-EC+MC-injected group represent the infarct areas (A) (mouse PECAM-1: red, Neu-N: blue. scale bar: 500 μm, ×5 magnification). B-C, Quantitative analysis of the infarct area (5 groups) in the striatum (B) and the infarct volume in the saline- and hES-EC+MC-inejcted group (C) on day 28 after MCAo.* *P *< 0.05. D-E, Representative fluorescent photographs on day 14 after MCAo and quantification of ss-DNA^+ ^cells in the ischemic penumbral area in the saline- and hES-EC+MC-injected group. (ss-DNA: green, Neu-N: blue. Scale bar:100 μm, ×20 magnification. *P < 0.05). F, Assessment of recovery of impaired motor function by quantification of the time from the start of the exercise until collapse on an accelerating rota-rod just before the experiments (= day 0) and on day 7 and 28 after MCAo. * *P *< 0.05.

There was no significant difference in the infarct area in the striatum on day 28 after MCAo between the saline- (1.372 ± 0.041 mm^2^: n = 10) and the hMNC- (1.438 ± 0.084 mm^2^: n = 10) injected groups. The infarct area in the hES-EC- (1.308 ± 0.094 mm^2^: n = 6) or the hES-MC- (1.249 ± 0.047 mm^2^: n = 6) injected group showed a tendency to decrease. A significant decrease in the infarct area was observed in the hES-EC+MC-injected group (1.167 ± 0.085 mm^2^: n = 9, *P *< 0.05) compared to the saline- and hMNCs-injected groups (Figure 6A, B). We also confined that the infarct volume was significantly reduced in the hES-EC+MC-injected group on day 28 after MCAo, compared to the saline-injected group (hES-EC+MC = 1.475 ± 0.083 mm^3^: n = 9, saline = 1.736 ± 0.057 mm^3^: n = 11, *P *< 0.05) (Figure 6C). On day 14 after MCAo, the number of ss-DNA^+ ^cells in the ischemic penumbral area in the hES-EC+MC-injected group (17.8 ± 2.5/mm^2^: n = 5, P < 0.05) significantly decreased compared to that of the saline-injected group (43.5 ± 5.4/mm^2^: n = 5) (Figure 6D, E).

### Assessment of recovery of impaired motor function after MCAo

We estimated the exercise time by the rota-rod to evaluate the recovery of impaired motor function. Just before the experiment (day0) and on day 7 after MCAo, there was no significant difference of the exercise time in the 5 groups. Even on day 28 after MCAo, significant recovery of impaired motor function was not detected in the hES-EC- (31.2 ± 0.8 seconds, n = 7) or the hES-MC- (30.8 ± 0.7 seconds, n = 7) injected group, compared to that of the saline- (29.5 ± 1.2 seconds, n = 12) or hMNC- (30.1 ± 0.8 seconds, n = 15) injected group. On the other hand, we observed the improvement in the hES-EC+MC-injected group on day 28 after MCAo (33.1 ± 1.3 seconds, n = 13 vs saline or hMNC group: *P *< 0.05) (Figure 6F).

### Expression of angiogenic factors in human ES cell derived MCs

**Figure 7:**
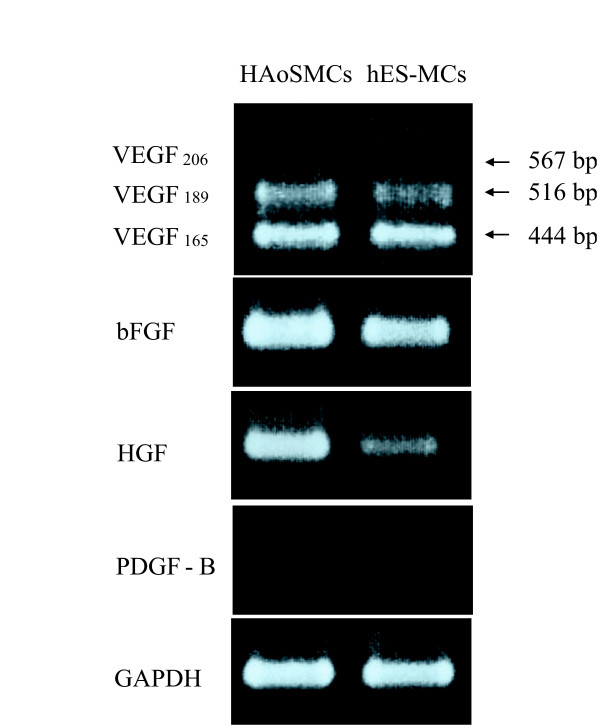
RT-PCR analysis of mRNA expression of VEGF, bFGF, HGF, and PDGF-B in hAoSMCs and hES-MCs. bp indicates base pair.

We investigated whether the transplanted hES-MCs produced major angiogenic factors such as VEGF, bFGF, HGF and PDGF-BB. Reverse transcription-polymerase chain reaction (RT-PCR) analysis detected mRNA expression of VEGF165, VEGF189, bFGF and HGF in MCs as well as hAoSMCs (Figure 7). In addition, we measured the protein concentration of these angiogenic factors in culture media of hES-MCs by enzyme-linked immunosorbent assay (ELISA). However, the concentration of all factors did not reach the detectable level as follows; the concentration of VEGF, bFGF or HGF was lower than 20 pg/ml, 10 pg/ml, or 0.3 ng/ml.

## Discussion

The findings reported here demonstrate that the transplantation of vascular cells, ECs and MCs derived from human ES cells, to the ischemic brain significantly promoted vascular regeneration in the infarct area and consequently contributed to neurological recovery after cerebral ischemia.

It was reported that in animal stroke models, the transplantation of human bone marrow stromal cells, which secrete basic fibroblast growth factor (bFGF) [16] and vascular endothelial growth factor (VEGF) [17], activates the endogenous expression of bFGF, VEGF and VEGFR2, and consequently promotes endogenous angiogenesis, while very few transplanted cells were incorporated into the host circulation [3]. Human CD34^+ ^cells isolated from umbilical cord blood were found to be capable of secreting several angiogenic factors, including VEGF, bFGF and hepatocyte growth factor (HGF) [18] and administration of these CD34^+ ^cells after cerebral ischemia was shown to promote endogenous angiogenesis mainly due to the supply of these angiogenic factors [5]. Bone marrow mononuclear cells containing small number of EPCs participated in neovascularization after focal cerebral ischemia in mice [4] or patients with limb ischemia [19]. However, Rehamn et al. demonstrated that EPCs, which were positive for acLDL and ulex-lectin, have little ability to proliferate and could release several angiogenic growth factors, i.e., VEGF, HGF and G-CSF [20]. Therefore, angiogenic effects induced by the transplantation of EPCs might be partially considered to be attributed to their growth factor secretion.

In contrast, ES cells with pluripotency and self-renewal are highlighted as a promising cell source for regeneration medicine. We have demonstrated that ECs- and MCs-derived from human ES cells could have a high ability of proliferation and be successfully expanded in large scale for the cell source of therapeutic vasculogenesis.

In the focal stroke model, endogenous angiogenesis in the ischemic area increased partially via the promotion of the expression of VEGF and bFGF in stroke areas [3], and in the present study, the increase of vascular density in saline-injected group on day 28 after MCAo was actually observed. The finding that there was no significant difference in CBF or vascular density between saline- and hMNCs-injected groups indicated that the effects induced by cell transplantation itself, such as the inflammatory reaction or embolic change, may have little or no influence on neovascularization after MCAo. Compared to the saline- or hMNCs-injected groups, CBF in the hES-EC-injected group increased significantly, while no significant increase in the number of mouse PECAM-1 positive cells was observed in the ischemic striatum on day 28 after MCAo. So, we consider that the transplanted hES-ECs detected in host capillaries could participate in neovascularization and make a partial contribution to functional blood vessels.

It is widely considered that during angiogenesis, the recruitment of periendothelial cells (MCs) toward endothelial cells sprouted from host capillaries promotes vascular stabilization and maturation [21-23]. We therefore assume that the increase in endogenous angiogenesis observed in the hES-MC-injected group in our study may have been partially due to a reduction in the retraction of newly-developed endothelial tubes and the promotion of vascular maturation via adequate MC coating.

Recent report demonstrated that endothelial cells derived from rhesus ES cells expressed von Willebrand factor (vWF), CD146 and CD34, but not CD31 and VE-cadherin by flow cytomerty and RT-PCR analyses [24]. Moreover, another report suggested that the cell surface VE-cadherin-negative populations derived during the differentiation procedure to vascular endothelial cells in cynomolgus monkey ES cells, which showed obvious cord-forming capacities and a uniform acetylated low-density lipoprotein (Ac-LDL)-uptaking activity, expressed VE-cadherin intracellularily. In addition, because RT-PCR analysis demonstrated the presence of the VE-cadherin message from the VE-cagherin-negative cells, they considered that these cells might be 'atypical' vascular endothelial cells [25]. Although, by reverse transcription-polymerase chain reaction (RT-PCR) analysis, we examined the mRNA expression of VE-cadherin in the hES-MCs to clarify whether the cell population was consisted of pure MCs or including 'atypical' ECs, the VE-cadherin message of the hES-MCs was not detected [see Additional file 1]. As shown in Figure 2H–I, the morphology of the hES-MCs was similar to hAoSMCs and all of the hES-MCs were positive for markers of mural cells as well as hAoSMCs. In the hES-MC-injected group, moreover, we could detect no human HLA-positive and αSMA-negative cells in the ischemic striatum, especially the host endothelial tubes. Therefore, we consider that the hES-MCs used for the transplantation were really pure MCs but not including 'atypical' ECs, and that the results observed in the hES-MC-injected group were brought by the transplantation of pure MCs itself.

The coordination of these beneficial effects on neovascularization of hES-ECs and hES-MCs could result in the increase in CBF and the marked promotion of vascular density in the ischemic striatum after the transplantation of hES-ECs+MCs. In the hES-EC+MC-injected group, the improvement in CBF was not seen to be as remarkable as that in the vascular density on day 28 after MCAo. Because the blood flow under the MCA, measured in our study, indicates the sum of both that in the ischemic striatum and that in the non-ischemic area, such as the cerebral cortex, we consider that the rate in the rise of CBF in the ipsilateral side might be underestimated.

We demonstrated that in the hES-MCs, RT-PCR analysis detected mRNA expression of some angiogenic factors, such as VEGF, bFGF and HGF, whereas the protein concentration of these factors in culture media was not enough to be detectable. Therefore, we consider that although the secretion of these angiogenic factors might have a possibility to affect the effect of hES-MCs transplantation, adequate MC coating might be more important for the promotion of endogenous angiogenesis after stroke, as observed in the hES-MC- or hES-EC+MC-injected group.

Moreover, in the hES-EC+MC-injected group, significant reduction of apoptotic cells in the ischemic core and infarct volume was observed. Even in a focal stroke model, it was suggested that greater than 80% of newly-formed neurons, which occurs in the subventricular zone of lateral ventricule or in the dentate gyrus of the hippocampus in the adult brain, died, most likely because of unfavorable environmental condition including lack of trophic support and exposure to toxic products from damaged tissues [26,27]. Thus, we assume that the marked promotion of neovascularization as seen in the hES-EC+MC-injected group could provide trophic support and remove toxic products to enhance survival of newly-formed neurons and consequently might promote neuroprotection in the ischemic striatum after stroke.

## Conclusion

We have demonstrated that ECs and MCs could be effectively differentiated from human ES cells and expanded on a large scale. Transplantation of these vascular cells markedly enhanced neovascularization in the ischemic brain and consequently promoted neuroprotection in a transient MCAo model. These finding suggest that vascular cells derived from human ES cells may have a potential to be a source for therapeutic vascular regeneration after stroke.

## Abbreviations

ES cells: Embryonic stem cells; VEGF-R2: vascular endothelial growth factor receptor type 2; ECs: endothelial cells; MCs: mural cells; hMNCs: human peripheral blood mononuclear cells; MCAo: middle cerebral artery occlusion; αSMA: alpha smooth muscle actin; hES-ECs+MCs: a mixture of ECs and MCs derived from human ES cells; hES-ECs: ECs derived from human ES cells; hES-MCs: MCs derived from human ES cells.

## Competing interests

The authors declare that they have no competing interests.

## Authors' contributions

NO wrote the manuscript, performed all experiments, and analyzed data. HI designed and revised the manuscript. MS, KY, DT, and HT participated the maintenance of human ES cell line (HES-3). KM participated the induction of middle cerebral artery occlusion (MCAo) in mice. KP, YF and NT analyzed data and performed statistics. MI and TS participated the maintenance of mice. KN designed and edited the manuscript. All authors read and approved the manuscript.

## Supplementary Material

Additional file 1 RT-PCR analysis of mRNA expression of VE-cadherin in hES-MCs, hES-ECs and HUVECs. Total cellular RNA was isolated from hES-MCs, hES-ECs and Human umbilical vein endothelial cells (HUVECs) with RNAeasy Mini Kit (QIAGEN K.K., Tokyo, Japan). The mRNA expression was analyzed with One Step RNA PCR Kit (Takara, Out, Japan). hES-ECs and HUVECs were used for positive controls. An initial 15-minute, 95°C hotstart was used, followed by cycles consisting of 1 minute denaturation at 94°C, 1 minute annealing, and 1 minute extension at 72°C. A 10-minute extension was done at 72°C after the final cycle. Thirty-five cycles were done for VE-cadherin. Oligonucleotide primer sequences, annealing temperature (Ta), and predicted product size of VE-cadherin were as follows; forward: 5'-ACGGGATGACCAAGTACAGC-3', reverse: 5'-ACACACTTTGGGCTGGTAGG-3', Ta: 58°C, product size: 597 base pair. mRNA expression of VE-cadherin was detected in the hES-ECs or HUVECs, but not in the hES-MCs. Click here for file
