# Supplementary figures and images for: Transplantation of vascular cells derived from human embryonic stem cells contributes to vascular regeneration after stroke in mice
Source: J Transl Med. 2008 Sep 30;6:54. doi: 10.1186/1479-5876-6-54 (PMC2567291; doi:10.1186/1479-5876-6-54)

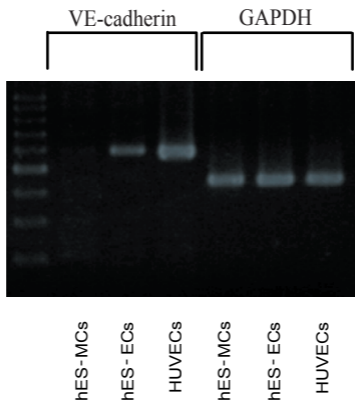

Supplement: Additional file 1 — RT-PCR analysis of mRNA expression of VE-cadherin in hES-MCs, hES-ECs and HUVECs. Total cellular RNA was isolated from hES-MCs, hES-ECs and Human umbilical vein endothelial cells (HUVECs) with RNAeasy Mini Kit (QIAGEN K.K., Tokyo, Japan). The mRNA expression was analyzed with One Step RNA PCR Kit (Takara, Out, Japan). hES-ECs and HUVECs were used for positive controls. An initial 15-minute, 95°C hotstart was used, followed by cycles consisting of 1 minute denaturation at 94°C, 1 minute annealing, and 1 minute extension at 72°C. A 10-minute extension was done at 72°C after the final cycle. Thirty-five cycles were done for VE-cadherin. Oligonucleotide primer sequences, annealing temperature (Ta), and predicted product size of VE-cadherin were as follows; forward: 5'-ACGGGATGACCAAGTACAGC-3', reverse: 5'-ACACACTTTGGGCTGGTAGG-3', Ta: 58°C, product size: 597 base pair. mRNA expression of VE-cadherin was detected in the hES-ECs or HUVECs, but not in the hES-MCs. [file 1479-5876-6-54-S1.pdf]
